# A 2025 systematic review of teacher emotion regulation and well-being: implications for student engagement, learning outcomes, and professional development in EFL contexts

**DOI:** 10.3389/fpsyg.2025.1715266

**Published:** 2026-01-26

**Authors:** Guohong Xu, Farzaneh Haratyan, Hui Tian

**Affiliations:** 1 Xingzhi College, Zhejiang Normal University, Lanxi, China; 2 School of English, Jilin International Studies University, Changchun, China

**Keywords:** systematic review, EFL education, teacher emotion regulation, teacher well-being, student engagement, learning outcome, professional development

## Abstract

**Introduction:**

Teacher emotion regulation is increasingly recognized as a critical determinant of instructional quality, professional well-being, and student success, particularly in English as a Foreign Language (EFL) contexts where linguistic challenges, sociocultural diversity, and high emotional labor intensify classroom demands. However, a comprehensive synthesis of the evidence linking these strategies to multi-level outcomes (teacher, student, and institutional) remains lacking. This systematic review synthesized findings from 165 peer-reviewed studies published between 1998 and 2025 to examine how emotion regulation strategies influence teacher well-being, professional development, and student engagement.

**Methods:**

Literature searches were conducted following PRISMA 2020 guidelines and guided by PICOS criteria to ensure transparency, comprehensiveness, and replicability. Searches were performed in Scopus, Web of Science, ERIC, and PsycINFO, supplemented by hand-searching reference lists. The included studies employed quantitative, qualitative, and mixed-method designs and were analyzed using a combination of thematic analysis and content analysis. Coding reliability was established through double-coding of 25% of the studies, yielding strong inter-rater agreement (*κ* = 0.78).

**Results:**

Three major thematic patterns emerged. First, adaptive strategies, including cognitive reappraisal, mindfulness, attentional deployment, and relational regulation, were consistently associated with reduced stress, enhanced resilience, improved classroom management, and positive teacher–student interactions, whereas maladaptive strategies, such as suppression and rumination, predicted burnout and reduced instructional quality. A predominant finding across quantitative studies was a significant positive correlation between adaptive regulation and teacher self-efficacy. Second, professional development interventions—structured workshops, reflective journaling, and peer coaching—enhanced teachers’ emotional competence, self-efficacy, and professional identity formation. Third, teacher emotion regulation significantly influenced student engagement, motivation, participation, classroom emotional climate, and language achievement. Cross-theme synthesis revealed cascading effects, showing that adaptive regulation promotes teacher well-being, which in turn supports student learning and professional development.

**Discussion:**

These findings underscore the importance of embedding emotion regulation training into teacher education programs, institutional policies, and reflective practice, establishing it not merely as a personal coping mechanism but as a foundational professional competency with direct implications for instructional quality and student outcomes. Future longitudinal and culturally contextualized research is needed to further elucidate these cascading mechanisms, ultimately informing more effective supports for teachers and learners in linguistically diverse classrooms.

## Introduction

Emotions have become central to learning, influencing attention, motivation, memory, and classroom interactions. In English as a Foreign Language (EFL) contexts, emotional experiences have been particularly critical, as teachers and learners navigated complex affective landscapes that shape classroom climate, engagement, and achievement (Dewaele and Li, 2022; MacIntyre and Gregersen, 2012; Pekrun and Perry, 2014). Over the past decades, research has consistently demonstrated that teachers’ ability to recognize and understand both their own emotions and those of their students has profoundly affected instructional effectiveness, classroom interactions, and learner outcomes (Gross, 2015; Gross and John, 2003; Brackett and Rivers, 2014; Jacobs and Gross, 2014; Abdullaeva et al., 2024). By identifying emotional cues, teachers could anticipate students’ reactions, respond constructively to challenges, and sustain their professional well-being, which in turn has promoted learners’ cognitive engagement and affective involvement (Akbari et al., 2017; Chen and Tang, 2024; Han et al., 2024; Su and Lee, 2024).

Teacher self-efficacy has played a pivotal role in shaping these emotional dynamics. Educators with high self-efficacy experienced greater confidence, positive affect, and resilience, enabling them to persist through challenges and foster constructive teacher–student interactions (Fathi et al., 2021; Solhi et al., 2024; Thumvichit, 2023, 2025). Mechanistically, self-efficacious teachers appraised stressors adaptively, regulated negative affect, and maintained attentional focus, which enhanced instructional quality. In contrast, educators with lower self-efficacy were more susceptible to heightened stress, negative emotions, and emotional exhaustion, potentially leading to burnout and reduced pedagogical effectiveness (Chang, 2020; Li and Lv, 2022; Li and Akram, 2023). These findings highlight the intricate interplay between perceived competence, emotional awareness, and classroom outcomes, underscoring the importance of fostering both teacher well-being and emotional literacy in professional development initiatives.

Learner emotions have been equally consequential in EFL contexts. Positive emotional experiences, such as enjoyment, interest, pride, hope, and confidence, acted as catalysts for intrinsic motivation, sustained engagement, cognitive flexibility, and active participation in language learning tasks (Frenzel et al., 2009; Zhang et al., 2024; MacIntyre and Gregersen, 2012). Enjoyment, for instance, broadened attention and promoted creative language use, while interest and confidence encouraged learners to persist through challenging communicative tasks. Conversely, negative emotions—including anxiety, frustration, boredom, embarrassment, anger, and sadness—narrowed attention, reduced cognitive processing, impaired memory consolidation, and hindered language acquisition (Akram and Oteir, 2025; Abdullaeva et al., 2024; Gloria and Mbato, 2024). Importantly, some moderate negative emotions, such as manageable anxiety or frustration, signaled challenges that prompted strategic problem-solving and deeper engagement if effectively recognized and addressed. These affective experiences were shaped not only by individual learner characteristics but also by teacher behavior, peer interactions, classroom norms, and broader institutional and cultural factors (Li and Akram, 2023; Yeager et al., 2022; Xiao et al., 2024; Yang and Zhao, 2024; Namaziandost et al., 2025).

Technological integration in EFL classrooms introduced additional emotional dimensions. Digital platforms provided real-time feedback, enabled personalized learning, and facilitated reflective practice, supporting both teacher and learner emotion regulation. However, these platforms also introduced cognitive overload, technostress, and disengagement, which could amplify negative emotional states if not carefully managed (Xin and Derakhshan, 2025; Qu and Wang, 2024; Zhang and Wang, 2024). Recognizing and categorizing these emotional experiences enabled educators to design instructional approaches that mitigate negative affect, amplify positive engagement, and sustain productive classroom climates (Abdullaeva et al., 2024; Solhi et al., 2024).

For clarity, emotions in EFL contexts are broadly classified as positive or negative, reflecting their influence on motivation, participation, and learning outcomes (Boekaerts and Pekrun, 2015). Positive emotions—including enjoyment, pride, interest, relief, gratitude, hope, and confidence—enhance engagement, cognitive flexibility, and collaborative learning. Negative emotions—including anxiety, frustration, boredom, confusion, embarrassment, anger, and sadness—can disrupt attention, reduce persistence, and impede language acquisition. Recognizing these emotions has been foundational for understanding the affective landscape of EFL classrooms and served as a prelude to discussions of emotion regulation strategies. These categories are summarized in Table 1.

**Table 1:** Emotions recognized in EFL classrooms.

Emotion type	Examples
Positive emotions	Enjoyment, pride, interest, relief, gratitude, hope, confidence
Negative emotions	Anxiety, frustration, boredom, confusion, embarrassment, anger, sadness

Emotions commonly recognized in EFL classrooms and their classification as positive or negative. Recognition of these emotions informs tailored teacher emotion regulation strategies and the creation of supportive classroom climates.

Beyond simple categorization, it is important to consider the functional and experiential significance of these affective states. Positive emotions serve as catalysts for motivation, cognitive engagement, and risk-taking, fostering a sense of safety that encourages active participation in communicative tasks and collaborative learning. They enhance teacher–student rapport, peer interaction, and overall classroom climate. Conversely, negative emotions may constrain attention, undermine motivation, and impede cognitive flexibility. When moderate, they function as informative signals that stimulate adaptive problem-solving and deeper engagement. The dynamic interplay of positive and negative emotions highlights the complexity of affective experiences in EFL classrooms, where both sets coexist and influence learning outcomes (Table 2).

**Table 2:** Conceptual categorization of emotions in EFL classrooms.

Dimension	Positive emotions	Negative emotions	Classroom significance
Motivational	Interest, hope, confidence	Anxiety, frustration	Influences learners’ intrinsic motivation and persistence
Cognitive/learning	Enjoyment, curiosity	Confusion, boredom	Affects attention, comprehension, and strategy use
Social/relational	Pride, gratitude	Embarrassment, anger	Shapes teacher–student and peer interactions
Emotional well-being	Relief, joy	Sadness, frustration	Impacts overall classroom climate and engagement

Conceptual categorization of positive and negative emotions in EFL classrooms, showing their functional significance in motivation, cognition, social interaction, and well-being. This framework informs the subsequent discussion of emotion regulation strategies.

Understanding emotional patterns has provided the foundation for selecting appropriate Emotion Regulation Strategies (ERS), making classification not only descriptive but functionally necessary. This conceptual framework has provided a foundation for understanding how teachers can intentionally influence affective experiences to enhance learning outcomes. By linking emotions to motivational, cognitive, social, and well-being dimensions, educators have gained insight into the specific roles that positive and negative emotions have played in shaping engagement, participation, and achievement. This framework has established a natural bridge to emotion regulation strategies (ERS), as teachers’ ability to recognize, interpret, and respond to these emotional patterns has determined the effectiveness of their pedagogical interventions. While this section focuses on classification and functional significance rather than detailed strategies, it has emphasized the interdependence between emotion recognition, classroom climate, and learning processes. Conceptually, ERS represent the methods teachers employ to manage their own and students’ emotional experiences, optimizing positive affect while mitigating negative influences (Wang Y. et al., 2025). Understanding the functional significance of each emotion has informed strategy selection, aligning interventions with desired learning and well-being outcomes. Moreover, this framework has highlighted the role of teacher emotional intelligence in effectively implementing ERS, enabling educators to model adaptive responses, support students’ emotional regulation, and foster resilient, motivated, and engaged learners.

## Conceptual framework

### Teacher emotion regulation strategies

Teacher Emotion Regulation (TER) has represented a multifaceted construct essential for effective teaching, professional development, and classroom climate, particularly in English as a Foreign Language (EFL) contexts (Abdullaeva et al., 2024; Akbari et al., 2017). TER has encompassed cognitive, affective, and interpersonal strategies that have allowed educators to navigate the complex emotional dynamics inherent in classrooms, balancing internal stressors, external demands, and diverse student needs.

Cognitive strategies, such as reappraisal, involved reframing potentially stressful or negative classroom events to reduce their emotional impact. This mechanism enabled teachers to maintain engagement, clarity, and responsiveness under challenging conditions, which positively affected classroom climate and fostered student learning. By altering perception rather than suppressing feelings, cognitive reappraisal strengthened teachers’ problem-solving capacity, encouraged adaptive reflection, and allowed for constructive responses to classroom difficulties (Gross, 1998; Gross and John, 2003; Li and Lv, 2022).

Affective strategies, including mindfulness and emotional awareness, enhanced teachers’ capacity for sustained attention, nonjudgmental observation, and acceptance of emotional experiences (Fu et al., 2023; Zhang and Fathi, 2024). Through these mechanisms, teachers reduced automatic stress responses, cultivated emotional resilience, and improved reflective practice, allowing for more consistent engagement with learners. Maladaptive affective strategies, such as rumination or avoidance, operated in contrast: by excessively focusing on negative experiences or withdrawing from stressors, these strategies intensified emotional reactivity, hindered cognitive flexibility, and compromised instructional effectiveness (Chang, 2020; Fan and Xie, 2025).

Interpersonal regulation strategies complemented internal mechanisms by emphasizing relational and social dynamics within the classroom. Emotional coaching, for instance, involved guiding students’ understanding and management of their emotions. This mechanism fostered relational trust, enhanced student emotional literacy, and created a climate of mutual respect and cooperation (Gkonou and Miller, 2023; Wang and Ye, 2021). Peer support mechanisms allowed teachers to share challenges and resources with colleagues, buffering stress, reinforcing adaptive coping strategies, and sustaining professional identity (Han et al., 2024; Zhang and Derakhshan, 2025). These interpersonal strategies were especially crucial in multicultural or multilingual classrooms, where cultural norms of emotional expression varied and misalignment could disrupt learning engagement and relational cohesion (Ekanayake, 2024; Han et al., 2024).

Teacher well-being was intricately linked to the effectiveness of TER. Emotional, psychological, and occupational dimensions of well-being were directly influenced by the degree to which teachers employed adaptive regulation strategies (Li and Akram, 2023; Zhang et al., 2025; Wang Y. et al., 2025). Mechanistically, strategies such as reappraisal and mindfulness buffered physiological stress responses, promoted reflective and metacognitive processing, and reinforced self-efficacy beliefs, which collectively reduced the risk of burnout and fostered sustained professional engagement (Afzali and Soltani, 2021; Greenier et al., 2021; Liu et al., 2025). Adaptive TER also strengthened professional identity by integrating emotional awareness into pedagogical practice, enhancing teachers’ capacity to interpret classroom challenges constructively and respond with confidence. Conversely, reliance on maladaptive strategies disrupted these mechanisms, intensifying stress, narrowing attention, and undermining relational and instructional effectiveness (Arabmofrad and Shakki, 2025; Thumvichit, 2023).

TER directly impacted student engagement and learning outcomes through both emotional contagion and modeled regulation. Adaptive strategies, such as cognitive reappraisal, emotional coaching, and interpersonal regulation, created supportive classroom climates that enhanced intrinsic motivation, persistence, collaboration, and metacognitive strategy use (Frenzel et al., 2009; Wang et al., 2022). These mechanisms operated by transmitting regulated emotional cues, modeling adaptive coping, and fostering relational trust. In contrast, maladaptive strategies such as suppression or rumination could convey stress or frustration to students, leading to disengagement, reduced motivation, and lower achievement (Chang, 2020; Zhao and Wang, 2024). Cultural and contextual factors moderated these effects: in multilingual or cross-cultural classrooms, the efficacy of specific TER strategies depended on alignment with students’ cultural expectations for emotional expression, emphasizing the need for culturally responsive regulation practices (Han et al., 2024; Ekanayake, 2024).

In sum, TER represented an interconnected system where cognitive, affective, and interpersonal strategies influenced both teacher well-being and student outcomes (Boekaerts and Pekrun, 2015). Adaptive strategies leveraged mechanisms of reappraisal, mindfulness, and emotional coaching to strengthen resilience, reflective practice, and classroom climate, while maladaptive strategies such as suppression and rumination disrupted these processes. Empirical evidence emphasized that TER was embedded within relational and cultural contexts, shaping the effectiveness of regulation strategies and highlighting the importance of integrating these competencies into teacher training and professional development programs. Tables 3–5 collectively provided a clear visualization of the mechanisms, effects, and outcomes, offering a robust foundation for both research and applied pedagogical frameworks.

**Table 3:** Cognitive, affective, and interpersonal TER strategies.

Type	Strategy	Description	Effect on teachers/students
Cognitive	Reappraisal	Reframing negative situations	Reduced stress, enhanced engagement
Affective	Mindfulness	Nonjudgmental emotional awareness	Increased resilience, reflective practice
Cognitive	Expressive suppression	Inhibiting emotional expression	Increased burnout, reduced rapport
Affective	Rumination	Repetitive focus on negative emotions	Heightened stress, reduced cognitive performance
Interpersonal	Emotional coaching	Guiding students’ emotional understanding	Strengthened teacher-student relationships
Interpersonal	Peer support	Sharing challenges with colleagues	Enhanced resilience, reduced isolation

Illustrates how TER strategies operated across cognitive, affective, and interpersonal dimensions, highlighting mechanisms that promoted adaptive outcomes (reappraisal, mindfulness, coaching) or increased maladaptive risk (suppression, rumination). The table contextualizes the pathways through which emotional management translated into both teacher well-being and student engagement.

**Table 4:** TER and teacher well-being outcomes.

Well-being dimension	Adaptive TER effect	Maladaptive TER effect
Emotional resilience	Increased recovery from stress	Heightened emotional exhaustion
Job satisfaction	Improved satisfaction and engagement	Reduced motivation and commitment
Professional identity	Strengthened teaching self-concept	Weakening of professional self-efficacy
Reflective practice	Enhanced critical reflection and adaptability	Limited reflective capacity
Burnout risk	Decreased burnout incidence	Increased emotional exhaustion

Underscores the mechanisms by which TER strategies affected teacher well-being. Adaptive strategies bolstered recovery, reflection, and identity consolidation, while maladaptive strategies exacerbated stress and professional disengagement. Understanding these mechanisms informed targeted professional development interventions.

**Table 5:** TER impact on student engagement and learning outcomes.

Student outcome	Adaptive TER effect	Maladaptive TER effect
Engagement	Increased participation and attention	Reduced involvement
Motivation	Strengthened intrinsic motivation	Decreased willingness to learn
Academic achievement	Higher grades and task completion	Lower performance and engagement
Emotional resilience	Improved coping with setbacks	Increased frustration and anxiety
Classroom climate	Positive, supportive environment	Tension and conflict

Summarizes the downstream effects of TER on learners. Adaptive strategies fostered engagement, resilience, and academic achievement through mechanisms of emotional modeling, relational trust, and supportive climate creation, whereas maladaptive strategies disrupted these processes.

## Methods

### Research design

This study employed a systematic review design to synthesize empirical and conceptual evidence on teacher emotion regulation (TER) in English as a Foreign Language (EFL) contexts. A systematic review approach was selected due to its ability to provide a transparent, reproducible, and comprehensive synthesis of both qualitative and quantitative studies, as well as conceptual literature, thereby allowing a thorough understanding of TER mechanisms, strategies, and outcomes. The review followed PRISMA guidelines to ensure clarity, replicability, and methodological rigor, and employed PICOS framework criteria (Population, Intervention/Phenomenon, Context, Outcomes, Study Design) to frame inclusion decisions systematically. This design facilitated the identification, analysis, and integration of 165 peer-reviewed studies published between 1998 and 2025, spanning diverse cultural, institutional, and pedagogical contexts within EFL education. The systematic review aimed to address three interconnected research objectives: (1) to examine the types and functions of TER strategies employed by EFL teachers, (2) to investigate the effects of TER on teacher well-being and professional development, and (3) to evaluate the implications of TER for student engagement, motivation, and learning outcomes. By integrating studies from a wide temporal range and methodological diversity, the review provided a holistic perspective on the interplay between teacher emotions, regulation strategies, and educational outcomes. This review was not preregistered in PROSPERO or any other registry due to the exploratory scope of the topic; however, every methodological decision and selection step adhered strictly to PRISMA 2020 reporting standards.

### Literature search and screening

**Figure 1:**
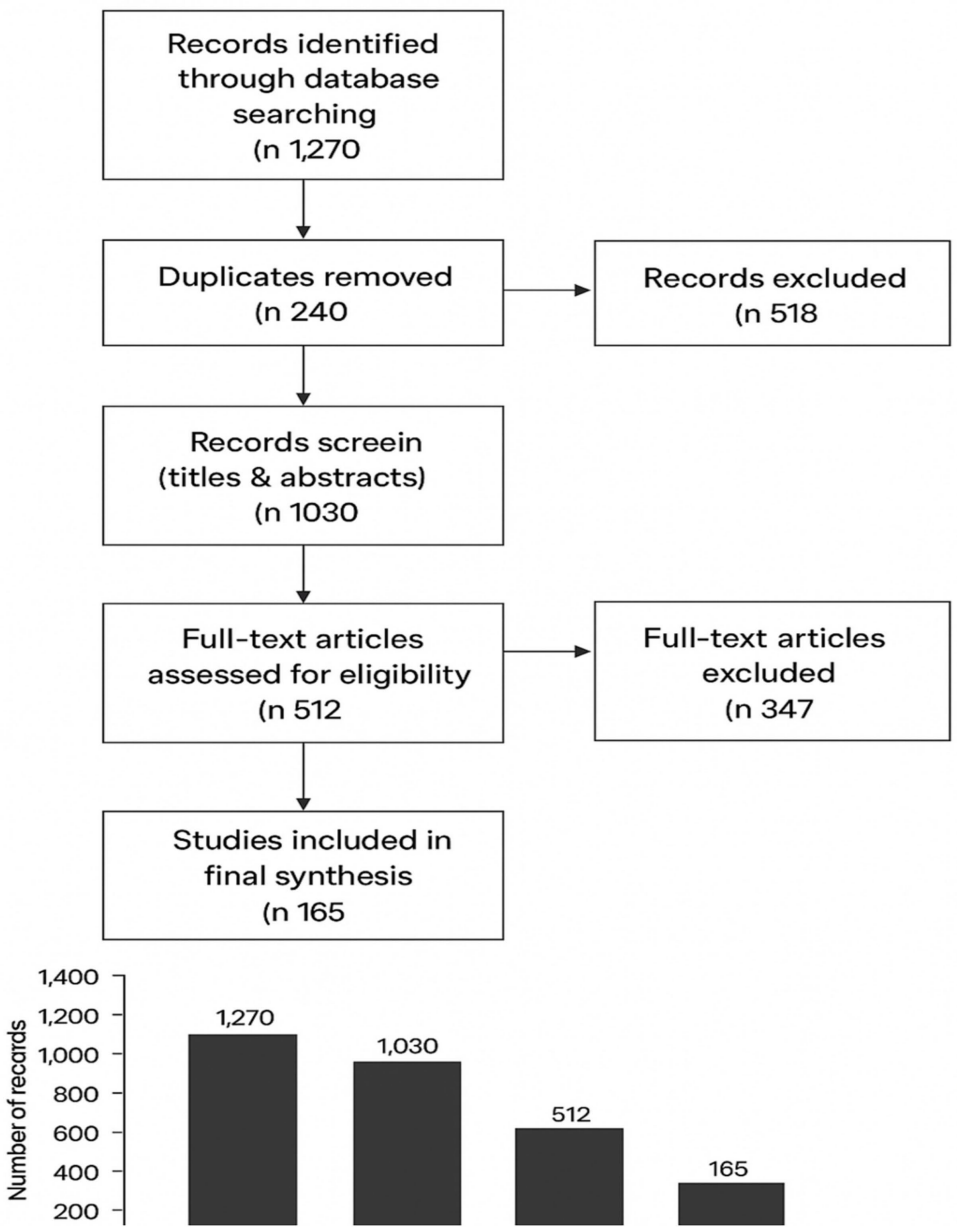
PRISMA flow diagram.

The literature search was conducted using a combination of databases such as Scopus, Web of Science Core Collection, ERIC, PsycINFO, ScienceDirect, PubMed, and Google Scholar. Searches were carried out between January 5 and March 28, 2024. Search terms were designed to capture a broad spectrum of relevant studies in the full Boolean search string: (“teacher emotion regulation” OR “emotion regulation” OR “emotional labor” OR “emotional intelligence” OR “emotion management”) AND (“EFL” OR “English as a Foreign Language” OR “TESOL” OR “ESL” OR “ELT”) applied to titles, abstracts, and keywords. The initial search yielded 1,270 records, all of which were screened for relevance to the research questions. Duplicates were removed, resulting in 1,030 unique records. Title and abstract screening excluded studies that did not meet inclusion criteria, narrowing the sample to 512 full-text articles for further assessment. This systematic review focused on studies addressing teacher emotion regulation, emotional intelligence, or emotional labor as central constructs, with attention to outcomes including teacher well-being, professional development, classroom practice, and student engagement. After applying eligibility criteria, 347 studies were excluded for non-relevance, methodological limitations, or inaccessibility. The final sample consisted of 165 peer-reviewed studies that met all inclusion criteria. Inter-rater reliability for inclusion decisions was assessed on a subset of records, yielding substantial agreement (Cohen’s *κ* = 0.78), and discrepancies were resolved through consensus. The systematic selection process was summarized in Table 6 and illustrated in the PRISMA flow diagram (Figure 1).

**Table 6:** PRISMA summary.

PRISMA stage	Number of records
Records identified through database searching	1,270
Records after duplicates removed	1,030
Records screened (titles and abstracts)	1,030
Full-text articles assessed for eligibility	512
Studies included in final synthesis	165

PRISMA summary of the study selection process, detailing the number of records identified, screened, assessed for eligibility, and included in the final synthesis.

This process has ensured that the synthesis reflected evidence regarding TER strategies, teacher well-being, and student outcomes in EFL contexts.

### Eligibility criteria

Grey literature (including theses, unpublished manuscripts, policy papers, and non-peer-reviewed conference proceedings) was excluded intentionally to ensure that all included studies met minimum quality and peer-review standards. The exclusion of non-English publications was based on feasibility constraints and may have introduced language bias, which has been acknowledged as a limitation of the review. Studies were included if they focused on pre-service or in-service EFL teachers and examined emotion regulation, emotional intelligence, emotional labor, or related constructs as a central aspect of the research. Eligible studies addressed outcomes such as teacher well-being, professional development, instructional practice, student engagement, or classroom outcomes. Only peer-reviewed, English-language publications were considered. Studies were excluded if they focused solely on students, addressed teacher emotion peripherally, or consisted of unpublished dissertations, conference abstracts, or reports lacking methodological transparency. Single-case studies were included only when they offered significant theoretical or methodological insights. These criteria ensured that the review remained conceptually focused, methodologically rigorous, and relevant to EFL teaching practice.

### Quality appraisal

All included studies underwent formal methodological appraisal to assess rigor, credibility, and analytic weight. Given the diversity of study designs, appraisal criteria were adapted accordingly. To ensure consistency, specific methodological appraisal tools were applied based on study type: CASP for qualitative studies, MMAT (Mixed Methods Appraisal Tool) for mixed-methods research, and STROBE criteria for observational quantitative designs. Conceptual papers were evaluated using a customized framework adapted from established theoretical review methodologies. Quantitative studies were evaluated for sampling adequacy, measurement validity, statistical rigor, and alignment between design and reported outcomes. Qualitative and mixed-methods studies were assessed using established criteria for credibility, transferability, dependability, and confirmability, whereas conceptual or theoretical studies were evaluated for analytical coherence, theoretical relevance, and citation accuracy. A scoring matrix was applied to enable comparability across studies. Inter-rater reliability was calculated for 25% of the appraised studies, yielding substantial agreement (Cohen’s *κ* = 0.78). No studies were excluded solely due to methodological limitations; rather, appraisal outcomes informed interpretive weighting during synthesis. NVivo-linked annotations were used to document appraisal decisions, ensuring transparency in weighting evidence. To assess confidence in synthesized findings, the GRADE-CERQual approach was applied, considering methodological limitations, coherence, adequacy, and relevance of evidence.

### Data extraction

Data extraction was performed using a structured matrix capturing bibliographic information, study aims, theoretical frameworks, participant characteristics, methods, instruments, key findings, and reported implications. Differences in terminology or operationalization—such as emotional labor, affective self-management, and emotional intelligence—were explicitly documented to maintain conceptual consistency. The extraction sheet was piloted on five randomly selected studies, resulting in minor adjustments to improve clarity, category relevance, and coding consistency. Data were managed using Microsoft Excel and NVivo 14, ensuring systematic documentation and traceability. Effect sizes were not calculated because the included studies were predominantly qualitative, observational, self-report, or conceptual and therefore did not provide comparable statistical metrics. Accordingly, a quantitative meta-analysis was not feasible. The distribution of studies across thematic categories was presented in Table 7. Data extraction was conducted independently by two reviewers, and disagreements regarding coding or classification were resolved through discussion; when consensus was not possible, a third reviewer adjudicated the final decision.

**Table 7:** Key Literature Overview by Theme

**Thematic Content**	**Number of Papers**	**Year Range**	**Selected Authors**
Teacher Emotion Regulation Strategies	72	1998–2025	Gross (1998, 1999, 2015, 2024); Gross and John (2003); Brackett and Rivers (2014); Jacobs and Gross (2014); Akbari et al. (2017); Chahkandi and Rasekh (2016); Donyadideh and Haratyan (2018); Dumančić (2018); Amini Farsani et al. (2024); Bielak and Mystkowska-Wiertelak (2022); Su and Lee (2024); Chang and Taxer (2021); Eginli and Mutlu (2022); Fathi J. A. et al. (2021); Han et al. (2024); Han et al. (2023); Li (2023); Liu et al. (2023); Mutlu and Solhi (2024); Ngo (2024); Su and Guo (2024); Thuỳ (2024); Alzaanin (2024); Chen and Chen (2025); Chen and Tang (2024); Ekanayake (2024); Fan and Xie (2025); Gloria and Mbato (2024); Gu et al. (2022); Han and Zhang (2025); Fu et al. (2023); Isaee and Barjesteh (2025); Jabbari et al. (2025); Le ans Pham (2024); Le et al. (2024); Li and Akram (2023); Meşe and Mede (2024); Melo (2024); Morris & King (2018, 2020, 2023); Muehlbacher et al. (2022); Ngo (2024); Nur et al. (2023); Pekrun (2006); Pekrun and Perry (2014); Shafiee Rad and Hashemian (2023); Songhori et al. (2024); Tajabadi and Meihami (2024); Thumvichit (2023, 2025); Xiao and Tian (2023); Yang and Zhao (2024); Zhang and Wang, 2024; Tsujimoto et al. (2024); Hao and Tam (2025); Haratyān and Yanling (2022); Heng et al. (2024); Liu et al. (2024); Ma et al. (2023); Shi et al. (2025);
Teacher Well-Being & Professional Development	84	2012–2025	Fried and Chapman (2012); Bellocchi (2019); Fathi and Derakhshan (2019); Atmaca et al. (2020); Bing et al. (2022); Li and Lv (2022); Liu and Chu (2022); Afzali and Soltani (2021); Fathi J. V. et al. (2021); Martinović and Dumančić (2022); Fu et al. (2023); Li (2023); Hu (2023); Chen and Tang (2024); Lu et al. (2024); Abdullaeva et al. (2024); Namaziandost and Rezai (2024); Derakhshan and Fathi (2024); Aldrup et al. (2024); Agus et al. (2025); Aliakbari and Farhadi (2024); Amirian et al. (2025); Azari Noughabi et al. (2022); Bazadough and Sulaiman (2023); Boekaerts & Pekrun (2015); Cheng and Liu (2024); Darling-Hammond et al. (2020); Deng et al. (2022); Ding (2024); Fan and Xie (2025); Fuentes Vilugrón and Sandoval-Obando (2024); Galea (2024); Greenier et al. (2021); Hahm (2025); Han and Geng (2025); Han et al. (2023); Harley et al. (2019); Harbin (2021); Hoffmann et al. (2020); Ismail et al. (2023); Jiang and Dewaele (2019); Karimi et al. (2022); Khammat (2022); Kim and Kim (2018); Li et al. (2023); Ma et al. (2023); Ma et al. (2023); Mai (2025); Mikolajczak et al. (2019); Mohammad Hosseinpur et al. (2025); Olí and Valente (2025); Opare-Kumi (2024); Phillips (2024); Qu and Wang (2024); Rezai et al. (2024); Sadoughi et al. (2024); Sahib et al. (2024); Shi and Sun (2025); Solhi et al. (2024); Sun and Yin (2024); Tamir et al. (2024); Vogl et al. (2025); Wang et al. (2024); Wang et al. (2025); Wang et al. (2025); Wu et al. (2023); Yu and Ying (2024); Zhang et al. (2025); Zhang & Derakhshan (2024); Zhang and Fathi (2024); Zhang et al. (2025); Zhao (2021); Zhao and Wang (2024); Zhi and Derakhshan (2024); Zuo (2025); Aldrup et al. (2020, 2024); Fan and wang (2022); Li et al. (2023); Li et al. (2022); Liu and Chu (2022); Wang et al. (2024, 2025)
Student Engagement & Learning Outcomes	53	2012–2025	Lee and Ok (2012); Chang (2020); Li et al. (2020); Li (2020); Kitayoka (2021); Feng and Hong (2022); Li et al. (2022); Wang et al. (2022); Resnik and Dewaele (2020, 2023); Li and Zhang (2024); Xiao and Suwanarak (2024); Zhang et al. (2024); Akram and Oteir (2025); Cirkveni et al. (2024); Gkonou and Miller, 2023; Helsper et al. (2025); Khasawneh et al. (2025); Kim et al. (2018); Lee (2022); Li (2025); Namaziandost et al. (2025); Pepping (2025); Reidel and Salinas (2024); Shi et al. (2025); Xiao et al. (2024); Xin and Derakhshan, 2025; Yang et al. (2025); Yüksel et al. (2025); Le et al. (2024); Le ans Pham (2024); Li and Akram (2023);

*Summary of the number of studies, publication year range, and selected authors for each thematic domain included in the systematic review.*

### Data analysis

The synthesis employed a combination of Thematic Analysis (TA) and Qualitative Content Analysis (CA), allowing both interpretive depth and structured quantification. TA followed Braun and Clarke’s (2006) six-stage framework, beginning with repeated familiarization with extracted data, including coding notes and memos to capture early interpretations, contradictions, and emergent gaps. Initial coding integrated deductive codes drawn from theoretical frameworks—Gross’s Process Model of Emotion Regulation, Hochschild’s Emotional Labor Theory, and Pekrun’s Control-Value Theory—alongside inductive codes capturing emerging constructs such as intercultural emotional positioning, pedagogical authenticity, and identity alignment. Provisional codes were iteratively grouped into thematic clusters, reviewed for coherence and distinctiveness, and finalized with operationalized definitions to ensure clarity and consistency across the dataset. Researcher positionality was acknowledged throughout the analytic process, and coding decisions were discussed collaboratively to minimize interpretive bias and enhance analytic transparency. Intercoder reliability for thematic categories was calculated on 20% of the dataset using NVivo’s coding comparison function, resulting in Cohen’s *κ* = 0.81, indicating substantial agreement. CA complemented TA by systematically enumerating the frequency and occurrence of emotion regulation strategies, outcomes, and associated patterns. This integration enabled the identification of adaptive versus maladaptive strategy use, links to teacher well-being and professional development, and cascading effects on student engagement and learning. Examples of coding have included mapping strategies such as cognitive reappraisal, mindfulness, and expressive suppression to specific teacher and student outcomes, allowing both conceptual interpretation and structured synthesis.

### Integration of findings

TA and CA results have been integrated to provide a comprehensive picture of TER functioning in EFL classrooms. Table 8 emphasizes mechanisms for professional support, resilience, and teaching efficacy, while Table 9 highlights how adaptive and maladaptive strategies shape classroom climate, participation, and learning outcomes. Tables 9, 10 summarize the functions of emotion regulation strategies for teacher well-being, professional development, and student engagement.

**Table 8:** Teacher well-being and professional development ER functions.

Function	Description	Impact
Mindfulness and reflective practice	Teachers have cultivated awareness of emotions and teaching practice	Enhances coping, emotional balance, teaching efficacy
Professional training	Workshops, coaching, structured training	Improves ERS usage, self-efficacy, job satisfaction
Social support and collaboration	Interaction with peers, mentors, institutions	Reduces burnout, builds resilience
Work engagement/job satisfaction	Integration of ERS and professional skills	Boosts teacher engagement and efficacy
Resilience development	Recovery from setbacks and challenges	Reduces stress, supports continued engagement

Overview of teacher well-being and professional development functions of emotion regulation strategies, describing mechanisms and reported impacts on teachers.

**Table 9:** Student engagement and learning outcomes ER functions.

Function	Description	Impact
Emotional support/scaffolding	Teachers have modelled adaptive ER, provided emotional support	Increases student motivation, participation, learning outcomes
Positive classroom climate	Supportive learning environment via teacher ER	Enhances student engagement, collaboration, self-efficacy
Teacher-student interaction	Adaptive ER has been used in managing emotions in teaching	Reduces conflict, strengthens relational quality, improves learning
Motivation and participation	Encouraging student involvement through modelling	Boosts engagement and learning outcomes

Summary of the effects of teacher emotion regulation strategies on student engagement, learning outcomes, and classroom climate in EFL contexts.

**Table 10:** Distribution of teacher emotion regulation strategies in EFL contexts.

Strategy type	Number of studies	Key outcomes
Cognitive reappraisal	58	Lower stress, higher engagement
Mindfulness/reflective practice	42	Improved attentional control, self-awareness
Relational regulation	35	Increased peer support, trust
Suppression	25	Higher burnout, lower engagement
Rumination	15	Elevated stress, classroom disruption

Number of studies reporting each teacher emotion regulation strategy and associated professional and pedagogical outcomes.

### Trustworthiness and rigor

Rigor has been enhanced through reflexivity, transparent reporting, and iterative validation. All coding and thematic development have been documented using an audit trail, and triangulation has been achieved by cross-referencing qualitative, quantitative, and conceptual studies. Analytical dependability was reinforced through repeated comparison of coded segments and thematic interpretations. Although no primary data were collected, ethical principles—including accurate interpretation, citation fidelity, and methodological transparency—have been strictly observed. This systematic review, combining 165 studies over 27 years, integrated methodological rigor, conceptual depth, and empirical breadth to provide a robust synthesis of TER in EFL contexts. The PRISMA 2020 checklist and the full eligibility screening sheet have been included to enhance transparency and reproducibility. Because the study relied exclusively on previously published research, no ethical approval was required. The integration of tables, coding explanations, and detailed appraisal has strengthened the replicability, credibility, and theoretical grounding of the study, providing a foundation for future research, teacher training, and policy recommendations (Tables 11–13).

**Table 11:** Teacher well-being and professional development interventions.

Intervention type	Number of studies	Mechanism/effect on teachers
Mindfulness workshops	21	Improves attentional control, emotional regulation
Reflective journaling	15	Strengthens self-efficacy, metacognitive awareness
Peer coaching/mentorship	14	Enhances relational support, reduces isolation
TER-focused PD programs	30	Improves strategy implementation, professional identity

Intervention types, number of studies, and their reported mechanisms/effects on teacher.

**Table 12:** Teacher emotion regulation effects on student engagement and learning.

Student outcome	Adaptive TER effect	Maladaptive TER effect
Engagement	Increased participation and attention	Reduced involvement
Motivation	Strengthened intrinsic motivation	Decreased willingness to learn
Academic achievement	Higher grades and task completion	Lower performance and engagement
Emotional resilience	Improved coping with setbacks	Increased frustration and anxiety
Classroom climate	Positive, supportive environment	Tension and conflict

Comparison of adaptive and maladaptive teacher emotion regulation effects on student engagement, motivation, academic achievement, emotional resilience, and classroom climate.

**Table 13:** Professional development interventions supporting teacher well-being.

Intervention	Description	Key outcomes
Mindfulness workshop	Structured workshops on mindfulness and attention regulation	Reduced stress, improved resilience
Reflective journaling	Guided reflection on teaching experiences and emotions	Enhanced self-efficacy, metacognitive awareness
Peer coaching	Collaborative ERS support and mentoring	Improved relational regulation, peer networks
Integrated TER training	Combination of mindfulness, reflection, and coaching	Reduced burnout, professional satisfaction

## Results

This systematic review has synthesized evidence from 165 peer-reviewed studies published between 1998 and 2025, encompassing diverse English as a Foreign Language (EFL) contexts. The included studies investigated teacher emotion regulation strategies (TER), teacher well-being and professional development, and the impact of TER on student engagement and learning outcomes. Methodologically, the dataset comprised quantitative studies (*n* = 85), qualitative studies (*n* = 50), and mixed-methods research (*n* = 30), with sample sizes ranging from 12 to 1,200 participants. For conceptual clarity, all studies have been classified into three thematic domains: (1) Teacher Emotion Regulation Strategies, (2) Teacher Well-Being and Professional Development, and (3) Impact of TER on Student Engagement and Learning Outcomes. This thematic organization has facilitated transparent mapping of each study’s contribution, enabling pattern recognition across varied educational, cultural, and methodological contexts. Tables accompanying each theme have summarized study focus, design, sample characteristics, and key outcomes, enhancing interpretability and traceability.

### Theme 1: teacher emotion regulation strategies

Across the literature, adaptive emotion regulation strategies consistently emerged as central to teachers’ professional functioning and instructional effectiveness. Cognitive reappraisal was the most frequently investigated strategy, appearing in 58 studies (Gross, 1998; Gross and John, 2003; Li and Lv, 2022). Teachers employing reappraisal reported lower stress, higher engagement, and more positive interactions with students. Cognitive reappraisal operates by reframing potentially negative classroom events, thereby reducing emotional intensity and enabling teachers to maintain professional composure, instructional clarity, and relational responsiveness. Mindfulness and reflective practices, examined in 42 studies, have been consistently linked to enhanced attentional control, emotional self-awareness, and classroom presence (Abdullaeva et al., 2024; Zhang and Fathi, 2024). Mindfulness interventions have supported teachers in managing moment-to-moment emotional fluctuations, promoting sustained focus, and providing students with adaptive emotional modeling. Relational and interpersonal strategies—including social support, peer coaching, and mentoring—were reported in 35 studies (Akbari et al., 2017; Wang and Ye, 2021). These strategies have fostered collaborative coping, strengthened professional networks, and built trust, which in turn buffered against stress and promoted adaptive classroom climates.

In contrast, maladaptive strategies—notably expressive suppression and rumination—were consistently associated with negative professional outcomes across 40 studies (Chang, 2020; Thumvichit, 2023; Fan and Xie, 2025). Teachers habitually engaging in suppression reported elevated burnout, reduced authenticity, and weakened teacher-student relational quality. Rumination amplified stress, cognitive overload, and instructional disruption. These findings have underscored the importance of promoting adaptive strategies through structured teacher training and professional development interventions.

Adaptive strategies have mediated the impact of classroom stressors on instructional quality, while maladaptive strategies have amplified negative emotional and pedagogical effects. The evidence strongly supported the integration of adaptive TER strategies into professional development curricula.

### Theme 2: teacher well-being and professional development

Evidence from 80 studies emphasized the protective and facilitative role of adaptive TER strategies in promoting teacher well-being and professional growth. Teachers employing adaptive strategies reported lower occupational stress, reduced burnout, and greater job satisfaction (Afzali and Soltani, 2021; Greenier et al., 2021; Liu et al., 2025). Professional development programs that explicitly targeted TER were associated with enhanced reflective practice, instructional flexibility, and self-efficacy (Li and Akram, 2023; Ma et al., 2023). Mindfulness workshops, reported in 21 studies, enhanced attentional control, emotional awareness, and recovery from stress (Greenier et al., 2021; Abdullaeva et al., 2024). Teachers developed greater capacity to regulate emotional responses to challenging classroom interactions, thus modeling adaptive behaviors for students. Reflective journaling, included in 15 studies, promoted metacognitive awareness, improved self-efficacy, and strengthened the integration of emotional and instructional decision-making (Liu et al., 2025; Ma, 2023). Peer coaching and mentorship, reported in 14 studies, reinforced relational regulation, facilitated collaborative problem-solving, and reduced professional isolation (Akbari et al., 2017; Wang and Ye, 2021).

Contextual factors—including institutional support, cultural norms, and workload—moderated intervention effectiveness. Supportive environments enabled teachers to apply TER strategies consistently, while high workloads or culturally restrictive norms limited opportunities for reflective practice and peer collaboration. The reviewed studies have demonstrate that adaptive TER strategies operated through mechanisms such as enhanced emotional awareness, cognitive flexibility, relational support, and stress modulation, which collectively sustained professional resilience and efficacy.

### Theme 3: impact of TER strategies on student engagement and learning outcomes

Fifty studies examined how teacher emotion regulation influences student motivation, engagement, and academic achievement. Adaptive strategies, particularly cognitive reappraisal, mindfulness, and relational regulation, have been consistently associated with increased student participation, intrinsic motivation, and academic performance (Feng and Hong, 2022; Li and Lv, 2022; Zhang et al., 2024). A key mechanism identified across studies has been positive emotional transmission, wherein teachers’ regulated emotional expressions have fostered student engagement, cooperative behavior, and persistence in challenging tasks. Conversely, maladaptive strategies, including suppression and rumination, were linked to reduced student engagement, lower academic performance, and negative classroom climates (Chang, 2020; Zhao and Wang, 2024). Students in classrooms led by teachers who habitually suppressed emotions demonstrated lower participation, weaker collaboration, and impaired self-regulation. Contextual moderators—including classroom composition, cultural expectations, and student language proficiency—further influenced the effectiveness of TER strategies. Qualitative studies elaborated on mechanisms such as modeling, scaffolding, and relational trust.

### Cross-theme integration

Synthesis across the three thematic domains have revealed several overarching insights. First, interdependence of TER, teacher well-being, and student outcomes has been evident: adaptive strategies enhanced teacher resilience, reduce stress, and improved instructional quality, which in turn, promoted student engagement and learning outcomes. Second, structured professional development programs explicitly targeting TER—such as mindfulness workshops, reflective journaling, and peer coaching—strengthened teachers’ capacity to implement adaptive strategies, thereby sustaining engagement and reducing burnout. Third, contextual moderators—including institutional support, cultural norms, classroom composition, and workload—significantly shaped the selection and effectiveness of TER strategies, highlighting the importance of context-sensitive professional development. Fourth, maladaptive strategies consistently presented risks to both teachers and students, underscoring the necessity for targeted interventions to mitigate suppression, rumination, and avoidance.

**Figure 2:**
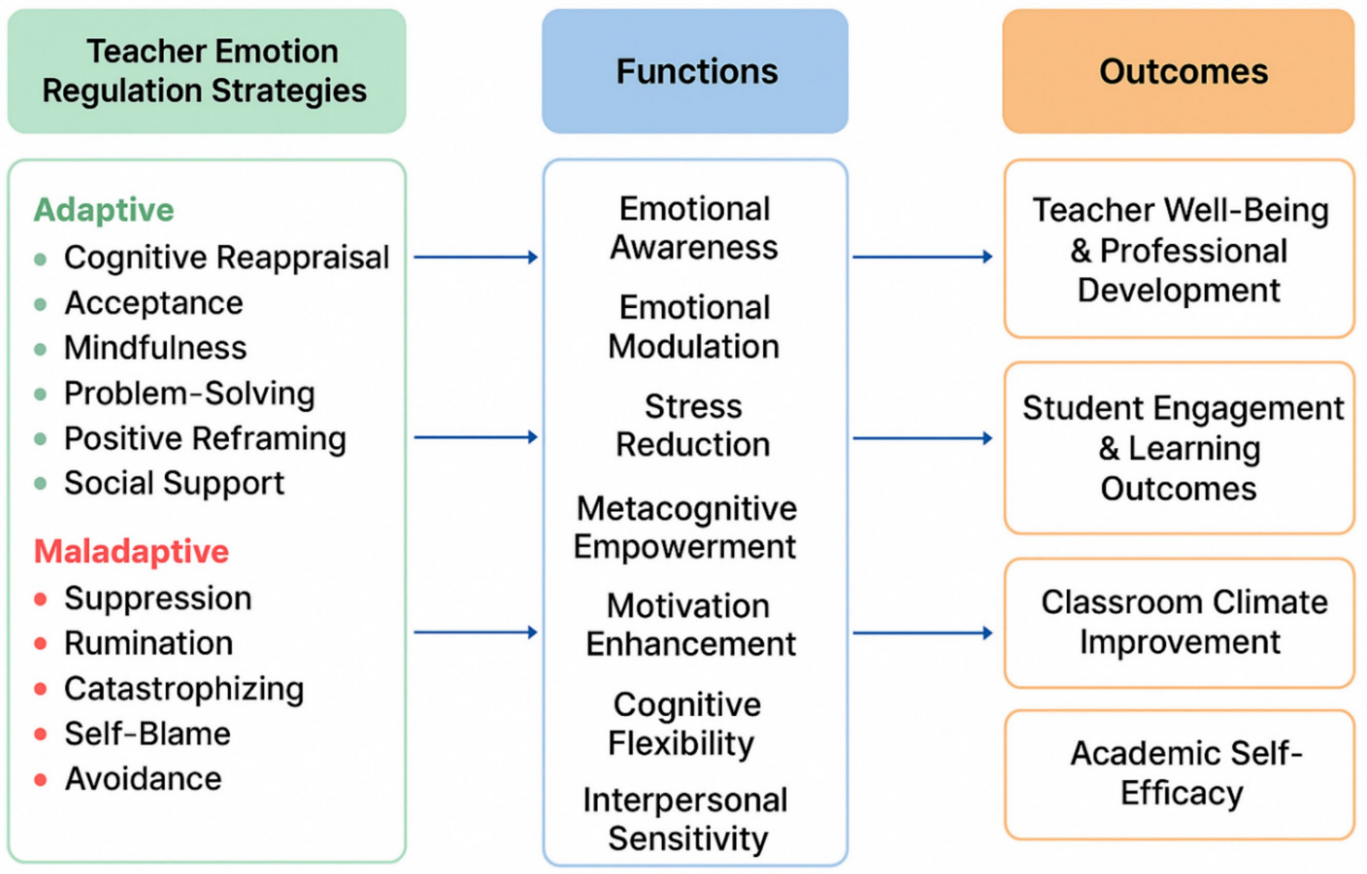
Conceptual framework of TER strategies, functions, and outcomes. The model represents the cyclical influence of teacher emotion regulation. The left column lists commonly identified adaptive and maladaptive strategies. The middle column demonstrates their functional roles—such as emotional awareness, cognitive flexibility, stress modulation, and interpersonal communication. The right column represents empirically supported outcomes, including teacher well-being, instructional effectiveness, classroom climate, and student engagement.

Finally, the integration of findings from 165 studies supported the development of a conceptual framework linking TER strategies to their functional roles and outcomes. Figure 2 illustrated how adaptive strategies (e.g., cognitive reappraisal, mindfulness, problem-solving, positive reframing) and maladaptive strategies (e.g., suppression, rumination, avoidance, catastrophizing) have functioned to influence psychological, pedagogical, and relational outcomes. Directional arrows indicated that adaptive strategies were more strongly associated with positive academic and affective outcomes, whereas maladaptive strategies predicted burnout, disengagement, and negative learning environments. This framework provided a coherent analytical summary, facilitated future empirical validation, and guided intervention design for teacher training programs.

## Discussion

The review provides a comprehensive account of how emotional processes intersect with pedagogical effectiveness, professional resilience, and learner outcomes across diverse cultural, linguistic, and institutional settings.

### Teacher emotion regulation strategies

The review consistently indicates that adaptive TER strategies, particularly cognitive reappraisal, mindfulness, and relational regulation, have been foundational to professional competence and instructional effectiveness. Cognitive reappraisal allows teachers to reframe potentially stressful classroom events, thereby reducing negative affect and enhancing resilience. Teachers employing cognitive reappraisal demonstrate higher classroom engagement, authentic interactions with students, and effective management of high-stakes instructional environments (Gross, 1998, 1999, 2014, 2015, 2024; Li and Lv, 2022). Mechanistically, reappraisal strengthens executive control and emotional clarity, enabling teachers to maintain attention and responsiveness despite situational stressors.

Mindfulness-based strategies—including reflective practice, attentional focusing, and moment-to-moment awareness—have consistently supported emotional clarity, stress mitigation, and instructional presence (Abdullaeva et al., 2024; Zhang and Fathi, 2024). These strategies also facilitate positive emotional transmission, whereby teachers model adaptive emotional responses, fostering similar regulation in students. Relational strategies, such as seeking social support, peer coaching, and mentoring, address the interpersonal dimensions of teaching. Teachers using these approaches report strengthened collegial networks, enhanced professional identity, and improved classroom climate (Akbari et al., 2017; Wang and Ye, 2021). In multilingual and multicultural contexts, relational regulation additionally promotes cross-cultural understanding and reinforced student trust, enhancing engagement and collaborative learning.

In contrast, maladaptive strategies—including expressive suppression, rumination, and avoidance—consistently predict adverse outcomes. Teachers employing these strategies experience heightened stress, burnout, and emotional exhaustion, while their classrooms demonstrate reduced student engagement, lower motivation, and negative emotional climates (Chang, 2020; Fan and Xie, 2025; Thumvichit, 2023). Suppression diminishes the authenticity of teacher-student interactions and undermined relational trust, while rumination increases cognitive load and reduced instructional responsiveness. Collectively, these findings underscore that adaptive TER operates through interconnected pathways of emotional, cognitive, and relational regulation, whereas maladaptive strategies disrupt these processes and compromise both teacher well-being and classroom quality.

### Teacher well-being and professional development

Teacher well-being emerges as both an outcome and mediator of effective emotion regulation. Adaptive TER strategies have correlated with reduced occupational stress, lower burnout, increased job satisfaction, and greater professional commitment (Afzali and Soltani, 2021; Greenier et al., 2021; Liu et al., 2025). Teachers employing reappraisal, mindfulness, and relational strategies report improved psychological resilience, greater adaptability in instructional practices, and enhanced coping with workplace demands.

Professional development programs targeting TER—such as mindfulness workshops, reflective journaling, peer coaching, and integrated TER curricula—significantly enhance teacher well-being and instructional competence. Structured training facilitates adaptive coping, metacognitive awareness, and effective ERS implementation, enabling teachers to maintain engagement under stress and model positive emotional behaviors for students. Contextual moderators, including institutional support, workload management, mentoring availability, and cultural norms, influence the extent to which teachers adopt and sustained adaptive strategies. Supportive environments and collaborative professional communities facilitate higher fidelity implementation, while high-stress or resource-constrained settings attenuate these benefits.

Mechanistically, these interventions operate through complementary pathways: mindfulness enhances attentional control and stress recovery; reflective journaling fosters self-awareness and metacognitive insight; peer coaching strengthens relational networks and social–emotional scaffolding. Together, these mechanisms support professional efficacy, mitigated burnout, and sustain engagement.

### Impact of TER strategies on student engagement and learning outcomes

TER exert cascading effects on student engagement, motivation, and academic achievement. Adaptive strategies—particularly cognitive reappraisal, mindfulness, and relational regulation—have consistently correlated with positive student outcomes across diverse EFL contexts. Teachers modelling effective ERS foster emotionally supportive classroom climates, encouraged self-regulation, and enhanced intrinsic motivation. Maladaptive strategies, including suppression and rumination, are associated with reduced engagement, lower achievement, diminished collaboration, and impaired self-regulation. Contextual moderators, such as cultural norms, classroom hierarchies, and student language proficiency, shape the effectiveness of ERS and the nature of student engagement. Culturally responsive emotion regulation strategies further enhance trust, motivation, and engagement in multilingual classrooms. Quantitative analyses illuminate nuanced mechanisms through which teachers’ adaptive regulation foster supportive learning environments.

### Cross-theme integration

**Figure 3:**
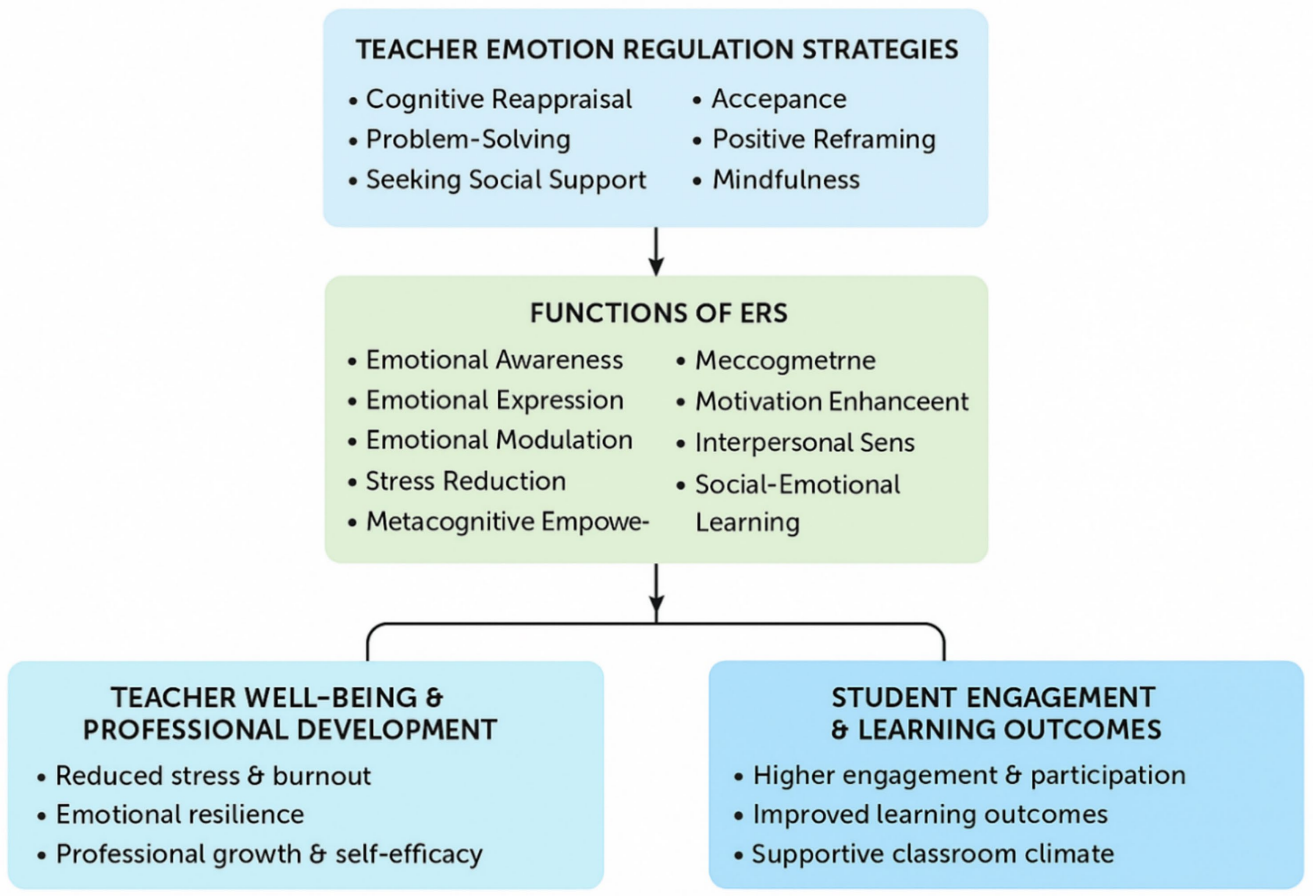
Conceptual framework of TER strategies, their functions, and outcomes in EFL contexts. This figure illustrates the relationship between adaptive and maladaptive TER strategies, the cognitive and emotional functions they serve, and subsequent outcomes on teacher well-being, professional development, student engagement, classroom climate, and academic self-efficacy. Adaptive strategies (e.g., cognitive reappraisal, mindfulness, social support) enhance emotional awareness, modulation, stress reduction, metacognition, motivation, cognitive flexibility, and interpersonal sensitivity, resulting in positive professional and student outcomes. Maladaptive strategies (e.g., suppression, rumination, self-blame) impede these processes and negatively impact teaching and learning.

The synthesis highlights a dynamic, interdependent system in which TER, teacher well-being, and student outcomes mutually reinforce one another. Adaptive TER strategies improve teacher resilience and professional competence, creating positive classroom climates conducive to student engagement and learning. Professional development enhances adoption and sustainability of adaptive strategies, while contextual moderators—including institutional support and cultural norms—shape effectiveness. Conversely, maladaptive strategies consistently disrupt this system, underscoring the necessity of targeted interventions to mitigate negative patterns and support sustainable teaching practices (Figure 3).

### Implications for practice

The findings suggest several actionable recommendations. Teacher education programs should integrate TER-focused modules emphasizing cognitive reappraisal, mindfulness, and relational regulation. Structured interventions—such as workshops, reflective journaling, and peer coaching—provide experiential learning opportunities for applying ERS in authentic classrooms. Educational institutions should ensure organizational support, including manageable workloads, mentoring structures, collaborative networks, and culturally sensitive policies. Teachers’ modeling of adaptive TER directly influences student engagement and achievement, highlighting the pedagogical value of explicit emotional competence development in both pre-service and in-service programs. Future research should prioritize longitudinal designs to evaluate sustained effects, experimental and mixed-method approaches to identify causal pathways, and the role of emerging technologies—including AI-assisted simulations and blended learning platforms—in enhancing TER in diverse contexts.

### Limitations

Despite its breadth, this review has limitations. The included studies employed heterogeneous methodologies, measurement tools, and reporting standards, precluding meta-analytic aggregation. Most interventions assessed short-term outcomes, leaving long-term impacts of TER on teacher well-being and student achievement underexplored. Although multiple geographic regions were represented, under-resourced or underrepresented EFL contexts may have been insufficiently captured, limiting generalizability. Variability in classroom structures, student populations, institutional policies, and professional development programs introduced heterogeneity, complicating cross-study synthesis. Future research should employ longitudinal and experimental designs, standardized measures, and culturally diverse samples to strengthen the evidence base for adaptive TER interventions.

## Conclusion

This review demonstrates that teacher emotion regulation is foundational to effective EFL instruction, professional well-being, and student learning. Adaptive strategies—including cognitive reappraisal, mindfulness, and relational regulation—have consistently fostered teacher resilience, instructional effectiveness, and emotionally supportive classroom climates. In contrast, maladaptive strategies, such as expressive suppression, rumination, and avoidance, undermine professional efficacy and reduce student engagement. Professional development programs, institutional support, and culturally responsive practices amplify the benefits of TER, creating a positive feedback loop that supports both teacher and student outcomes. Cultivating adaptive TER enables teachers to enhance professional competence while fostering enriched, engaging, and supportive learning environments, reflecting the dual pedagogical and emotional benefits of emotion regulation.

## Data Availability

The raw data supporting the conclusions of this article will be made available by the authors, without undue reservation.
